# Orientation work: caring for the relevance of research to social-environmental problems

**DOI:** 10.1080/09505431.2025.2531747

**Published:** 2025-07-21

**Authors:** Ruth Falkenberg, Lisa Sigl, Maximilian Fochler

**Affiliations:** a Research Platform Responsible Research and Innovation in Academic Practice, University of Vienna, Vienna, Austria; b Service Unit of Responsible Research Practices, TU Wien, Vienna, Austria; c Department of Science and Technology Studies, University of Vienna, Vienna, Austria; d Centre for Technology, Innovation and Culture (TIK), University of Oslo, Oslo, Norway

**Keywords:** Soil carbon, research directions, research governance, responsiveness, values, enabling conditions

## Abstract

Relevant research in a longer-term perspective is not result of a one-point intervention and re-direction of research to address social-environmental problems. Rather, for a research field to stay relevant to social-environmental issues, researchers and their communities need to continually engage in what we conceptualise as orientation work. Contrary to the notion of an invisible hand governing science in self-organised ways, the notion of orientation work offers a novel conceptual perspective on changes in research direction in the sense of caring for relevance, defined as *open-ended, responsive*, and *collective* process. Orientation work requires certain *conditions of possibility* and *attention to value-based questions.* The case of soil carbon research illustrates how orientation work can prevent research fields from getting stuck on paths that have (partly) lost their relevance. Slowly emerging in the 2000s, promises that carbon sequestration in soils could make substantial contributions to mitigate the climate crisis have generated much attention and, consequently, research funding and institutional support. Particularly with regard to responding to the climate crisis however, researchers have started to question the strong prioritisation of studying soil carbon within their research field, calling for reflections on its social-environmental relevance. Debates within soil carbon research provide evidence how orientation work on the level of research communities (besides other levels of research governance) can play an important role in addressing social-environmental problems.

## Introduction

In a world with limited resources and time, how do we know whether producing more knowledge on a topic is better than changing the orientation of research? The case of soil carbon research speaks to this question.

In the past two decades, soils and soil management have been identified as important for mitigating climate change, most importantly by sequestering carbon in soils. This has become visible in funding programmes, as well as initiatives by scientists and governments (‘4 per 1000’ initiative,^1^ EU mission ‘A Soil Deal for Europe’^2^). While soil researchers have contributed to developing these initiatives, they have also started scrutinising this idea, and have contributed to adapting and re-orienting the trajectories of soil research in response to experienced shortcomings. Based on an analysis of this dynamic in soil carbon research, we argue that collective engagement of research communities can help to orient research practices in ways that are better suited for addressing social-environmental problems.

It is nowadays broadly agreed that science has an essential role in addressing the multiple social-environmental crises we are facing. On the level of science policy for example this is reflected in calls for research to contribute to the UN’s Sustainable Development Goals, or to address ‘missions’ or ‘grand challenges’ (Bos et al., 2014; Ulnicane, 2016; Mazzucato, 2018; Kaltenbrunner, 2020). While some countries have included criteria such as relevance or impact in research evaluations (see, e.g. Neyland et al., 2019; Watermeyer and Chubb, 2019), it appears that so far most attempts to steer science towards social-environmental relevance have focused on research funding.

Yet, while available funding certainly influences how researchers orient their work (Gläser and Laudel, 2016), studies have shown that individual research groups working with such funding may find it difficult to interpret the often ‘symbolic but ultimately vague targets’ of the funding schemes (Kaltenbrunner, 2020, p. 19). Further, researchers may get confused over conflicting conceptions of relevance (Schikowitz, 2019), and experience tensions within the competitive academic career system (Fochler et al., 2016). Due to such dynamics, relevance and societal responsibility as can run the danger of being tick-boxed rather than seriously considered in actual research practices (Felt, 2017).

In this paper, we therefore draw attention to a different level of orienting research towards social-environmental relevance, namely that of research communities. Engaging perspectives that see relevance as open-ended and processual (Savransky, 2016; Klenk and Meehan, 2017) and feminist scholarship on care (Mol, 2008; Puig de la Bellacasa, 2011; Martin, Myers, and Viseu, 2015; Haraway, 2016), we ask: What role can collective and ongoing engagement on the level of research communities play in re-adjusting research paths in ways considered more relevant to social-environmental problems?

We explicitly inquire into the collective, not the individual, dimension of such continuous re-orientation towards relevance. Doing so, we are very much aware that in contemporary technoscience, research communities do not only come in the form of traditional disciplines or fields, but also as ‘emerging communities’ (Molyneux-Hodgson and Meyer, 2009), ‘scientific/intellectual movements’ (Frickel and Gross, 2005), or ‘interdisciplines’ (Frickel, 2004) that display heterogeneity on multiple levels (Kastenhofer and Molyneux-Hodgson, 2021).

Rather than stable entities, scientific communities often constitute ‘moving targets’ (Meyer and Molyneux-Hodgson, 2010), whose emergence and (temporal) stabilisation may be influenced not only by shared research repertoires (Ankeny and Leonelli, 2016) or particular technologies (Mody, 2011), but also by funding priorities or particular social-environmental problems and respective socio-technical promises about how to address them (Martin, Brown, and Kraft, 2008; Granjou and Arpin, 2015; Kastenhofer and Molyneux-Hodgson, 2021; Sigl, Falkenberg and Fochler, 2023). The latter factors, in particular, seem important in tying together the community of soil carbon research.

While research related to soil carbon has been going on for many decades, the soil carbon research community as analysed in this paper has largely formed since the 2000s, in relation to the essential role of soils as carbon sinks or sources in the climate crisis. The community is constantly changing and has grown to be rather broad and heterogeneous, including researchers from backgrounds such as traditional physico-chemical soil sciences, soil (microbial) ecology, ecosystem sciences, or modelling.

Drawing from interviews with soil carbon researchers, and in broader soil-related research, we inquire into the role of collective orientation work in guiding research practices towards social-environmental relevance. Using the case of soil carbon as an example, we suggest that research communities can support policy and funding shifts related to relevance, but also, that it can engender problematic dynamics if relevance is taken to be a relatively fixed and stable concern that is not continuously reflected upon. Bandwagon-like growth of research on a certain topic can, even if this topic is broadly considered very relevant, become rather disadvantageous if no ongoing care is taken of the relation between research questions and their relevance. Thus, we suggest that collective orientation work on the level of research communities can play an essential role in continuously re-adjusting research paths to addressing social-environmental problems.

By framing orientation work as care for relevance we argue that it is most powerful when approached as collective and value-based debate on what relevance can mean in a specific situation and at a specific point in time, and when it is flexible enough to respond to developments within and outside science. Yet, such care for relevance on the level of research communities also requires certain conditions of possibility. These are currently not necessarily given, as the academic system rather favours activities more immediately oriented towards the production of novel facts and paper-based output than practices related to developing relevance, such as synthesis or contributions to collective debates.

## Analytical perspectives

For our conceptual framework we combine two STS related perspectives. The first illustrates that both, current modes of research governance that enact academia primarily as a competitive market, and attempts to orient research towards societal relevance pay too little attention to the role of collective practices of research communities for epistemic re-orientations. Secondly, we draw from feminist scholarship to understand orientation work as care for relevance.

### Governing science – from competition on academic markets to orientation work on the level of research communities

Debates on the degree and the ways in which science can and should be steered have a long tradition in STS and science policy studies. An extreme position demands maximum autonomy for researchers: Michael Polanyi has been a vocal proponent of this opinion, arguing that ‘any authority which would undertake to direct the work of the scientist centrally would bring the progress of science virtually to a standstill’ (1962, 56). Only when left on its own, science would self-organise in the most efficient manner, as does – in his view – a market, with an ‘invisible hand’ (Polanyi, 1962, p. 56).

A similar idea of organising science in the form of a market can be found in contemporary neoliberal modes of research governance. However, rather than left on its own, academia is here actively *enacted* as a market, through the competitive allocation of funding or metrics-based evaluations (Lave et al., 2010; Burrows, 2012; Neyland et al., 2019). This actively engineered mode of governing through competition is, in turn, supposed to produce the most excellent or innovative science. While thus not explicitly directing research towards particular topics, such forms of governance nevertheless have important epistemic consequences, as they affect the ways in which researchers take decisions and orient their work (Fochler et al., 2016; Gläser and Laudel, 2016; Sigl, 2016; Falkenberg and Fochler, 2022; Pinel, 2022).

Next to governing research through competitiveness, another trend in light of current social-environmental crises is the more explicit alignment of research with public goals or with the stakeholder interests. Besides aligning research with such pre-set goals, more and more programmes aim to foster greater responsiveness to societal concerns more broadly and to integrate engagement with societal actors throughout processes of research and innovation (Bozeman and Sarewitz, 2011; Fisher and Schuurbiers, 2013; Owen et al., 2013; Stilgoe et al., 2013).

Importantly, however, both market-like modes of research governance and attempts to better align research with societal or stakeholder interests start from researchers and research groups as relevant entities of coordination. It is researchers or groups who are supposed to compete on the academic market (Polanyi, 1962; Neyland et al., 2019), to orient their work towards thematic funding calls (Kaltenbrunner, 2020), or to integrate societal responsiveness into their research agendas (Fisher and Schuurbiers, 2013; Stilgoe et al., 2013).

**Figure 1.**
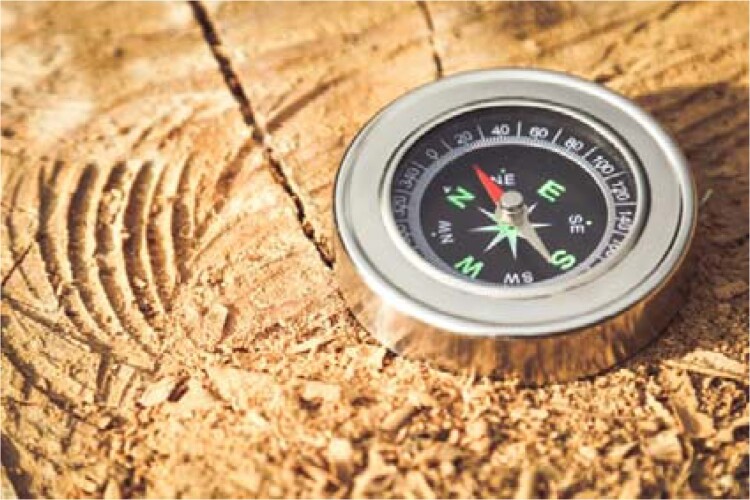
Compass (Picture by Jametlene Reskp, unsplash).

We argue that this leaves an attention gap on the potential role of collective engagement and coordination on the level of *research communities* when it comes to orienting research towards addressing social-environmental problems. We do not claim that research communities should have a more privileged role in guiding research agendas than societal actors. However, we suggest that research communities have expertise and insights to contribute to directing research and to care for its relevance which are too important to leave unconsidered. We will argue that orientation work encompasses practices aimed at both taking stock of the *present status* of research on a particular topic, and at debating and directing its *future development*. As we elaborate in the following section, we consider such work as essential for fostering a better *care for relevance* (Figure 1).

### Orientation work as care for relevance

We are inspired by work that highlights the processual character of relevance in research practices (Savransky, 2016; Klenk and Meehan, 2017). Feminist scholarship on care helps us to carve out important dimensions of orientation work that aims at re-orienting research towards relevance (Mol, 2008; Martin, Myers, and Viseu, 2015; Haraway, 2016).

First, a focus on care underlines the necessarily open-ended and *responsive* character of such work. Greater care for relevance can hardly be directed towards a fixed and unchanging goal, but must be an ongoing situated concern, a critical practice and a willingness to respond to the contingencies of a research process and the interferences it generates (Mol, 2008; Müller and Kenney, 2014; Martin et al., 2015). It requires ‘to attend persistently to new twists, turns, problems, frictions and complications’ (Mol, 2008, p. 76) and to ‘stay with the trouble’ (Haraway, 2016). In practice this means to continuously reflect, exchange, and try in practice what relevance may mean in a particular situation. This resonates with processual understandings of developing relevance (Savransky, 2016; Klenk and Meehan, 2017) that suggest the formulation of particular aspirational futures and an *active engagement* with potentially changing meanings of relevance throughout the research process, negotiated in relation to and together with research objects and participants.

Second, a care perspective implies that orientation work must be a *collective*, not an individual activity. Previous literature on approaches such as Responsible Research and Innovation (RRI) or midstream modulation has mostly focused on how individual researchers and smaller-scale research groups can integrate responsiveness to societal concerns into their practices (Fisher and Schuurbiers, 2013; Owen et al., 2013; Sigl et al., 2020). In contrast, we emphasise with Annemarie Mol (2008, 75) that in research as elsewhere, ‘[t]he task of establishing what ‘better’ [or, for our purpose, ‘more relevant’] might be involves collectives.’ Caring for relevance in knowledge production and re-orienting research practices accordingly must (also) happen on the level of a research community, in collective, open and ongoing exchange processes. This also means to invest in producing sociability, i.e. in creating and sustaining social relationships that allow for such collective work and value-based deliberation on what research trajectories are worth following or not (Puig de la Bellacasa, 2011).

The third dimension of a care perspective on orientation work addresses that caring for the relevance of research requires certain *conditions of possibility*, meaning that a person – or a collective – must not only have the willingness, but also the capacity to engage in practices of orientation work (Martin et al., 2015, Sigl and Fochler, 2025). If relevance is taken seriously, it also comes along with ‘demanding and complex temporal […] requirements’ (Savransky, 2016, p. 22), and actors must have the capacities to critically call into question the relevance of their practices, rather than being entrapped in a linear, progressivist productionism (Puig de la Bellacasa, 2015). This implies that orientation work as care for relevance must be seen and valued as the essential *work* that it is, rather than placing such activities back in favour of others considered more immediately productive. This resonates strongly with how feminist approaches to care pay attention to currently devalued issues and ways of upvaluing previously devalued kinds of work (cf. Puig de la Bellacasa, 2011).

Fourth, orientation work has a *value-based* and necessarily *normative* character. Recent studies have substantiated that broader shifts in research practices and epistemic commitments indeed entail collective re-valuations of what counts as good and relevant research (Granjou and Arpin, 2015; Falkenberg et al., 2024; Sigl et al., 2023; Sigl and Fochler, 2025). A care approach to discussing how this re-valuation takes place considers that decisions in research are necessarily value-based and take place within context-specific societal power relations that incentivise certain kinds of knowledge production while explicitly or implicitly disincentivising others (Rose, 1994). Caring for relevance thus requires to open and maintain time and space for negotiating the often implicit values that guide research in context-specific ways.

## Material and methods

This paper developed in the scope of the project *Valuing, Being, Knowing. Understanding the entanglements of valuation practices and subjectification processes in life science research.*^3^ In the project, we pursued a broader interest in how researchers take decisions in their work and negotiate the orientation of their research in relation to different ways of valuing science. Amongst others, we studied how researchers individually and collectively (decide to) re-orient their work towards addressing social-environmental problems, and inquired into conditions that facilitate such re-orientation. In order to do so we closely engaged with three research groups from the crop and soil sciences based in Austria. We conducted biographical and issue-centred interviews with them, organised workshops and group discussions, and occasionally observed their work in the lab and on the field.

In analysing our data, we found it striking how researchers often described their research to be strongly related to certain social-environmental problems but also expressed difficulties to concretely articulate this relevance in planning and conducting their actual research. We thus became interested in whether and how these individual narratives and struggles are linked to more collective debates within their research fields. An exploratory investigation showed that particularly the broader field of soil-related research seemed to have a longer history of reflecting the relevance of its research practices (see Sigl et al., 2023; Falkenberg et al. 2024).

Inquiring further into these developments we conducted a document analysis and 48 interviews with researchers from soil-related research. The document analysis included reports, scientific publications as well as comments and opinion papers that discuss the relevance of soil research since the 1980s. An initial set of analysed documents was identified from earlier analyses on debates about relevance in soil research (Sigl et al. 2023, Falkenberg et al. 2024), followed by a collection of documents that were recommended by interview partners (e.g. documents related to the ‘4 per 1000’ initiative) and scientific articles and editorials related to the role of soil carbon for solving social-environmental problems (e.g. Bradford et al. 2019, Chabbi et al. 2021). To understand such re-orientations in-depth, and in their respective societal and scientific contexts, the interviews started out with broadly discussing the development of soil science and soil-related research fields over time, how soil-related research has changed in relation to social-environmental problems, and what conditions scientists saw as (dis)advantageous for orienting their work in ways considered more relevant.

In a grounded theory-informed approach, we iteratively went back and forth between data analysis, literature, and further empirical data collection. One observation that arose in the process of data analysis was that researchers often spoke somewhat critically about bandwagon-like growth dynamics arising not only around research topics considered particularly innovative and cutting-edge, but also in areas considered very relevant to social-environmental problems. Soil carbon research was a particularly interesting case, as it was also subject to strong hype and growth dynamics and researchers had started questioning this hype, demanding a change in orientation of research on the relation between soil and climate change. We thus started focusing on the soil carbon case, with the aim to capture researchers’ perspectives on the strong growth dynamics and the changing degree of relevance of the work being done, as well as their changing opinions on what kinds of research best contribute to addressing social-environmental problems.

The sample of the 48 soil (carbon) researchers we interviewed is relatively broad and diverse, including all career stages (master students to principal investigators), and different orientations within the soil carbon community, representing the increasing breadth of soil-related research in general (e.g. soil microbial ecologists, soil scientists, soil macrofauna, soil rhizosphere, pedogenesis). The interviews were mostly carried out online via Zoom, which allowed a relatively broad geographic coverage (Europe, US, Australia and South America). Interviewees included, amongst others, more ‘traditional’ soil scientists working with mostly physico-chemical approaches, soil (microbial) ecologists, modellers, and researchers that adopted different inter  – and transdisciplinary approaches to soils in their work.

Interviewees were selected for their important contributions to soil (carbon) research (for example having editorial functions in relevant special issues and journals), or their prominent roles in policy-related contexts and initiatives related to soil (carbon) (e.g. contributing to relevant policy documents or initiatives). Based on initial research, the further selection of interviewees followed a snowballing approach, with further potential interview partners being suggested by earlier interview partners, or emerging as important actors during the document analysis.

In the following empirical analysis, we first describe how soil carbon research rose to prominence under the framing that soil carbon sequestration could play an important role in mitigating the climate crisis. We then outline the bandwagon-like growth dynamic that unfolded around the topic and illustrate researchers’ critiques of this growth, including different calls for re-considering the relevance of dominant research questions and approaches. Finally, we describe different practices of collective orientation work that researchers mentioned as necessary to guide soil carbon research in directions that seem better suited for addressing social-environmental problems.

### Soil carbon research rising to prominence

Soil carbon^4^ is not a new topic in soil research but has been studied for many decades, though with changing foci and in relation to different environmental problems (Smith et al. 2018). The currently strong focus on soil carbon research in relation to climate change slowly began to emerge in the 1990s. Yet, as different participants noted, it did initially not gain much traction and was seen by many scientists as rather unimportant and uninteresting.

This changed from the 2000s onwards, when soil carbon research gained prominence for different reasons: First, awareness was rising for the role of soils as carbon sinks and their potential in mitigating the climate crisis (see, e.g. McBratney et al., 2014). As one participant explained, ‘one of the big things that soils are of interest for is climate change, carbon sequestration – the potential ability to remove carbon dioxide from the atmosphere’ (R48).^5^ This growing attention led to a strong increase of available funding in the area, which – together with the opportunity to address an important social-environmental problem – seems to have attracted many researchers to work on the topic.

Second, in the early 2000s, novel technological possibilities opened up new perspectives. Particularly the field of soil microbiology is often described as especially important here, allowing ‘to study soil organic matter at the scale that is relevant, basically, microorganisms’ (R77). While microbes play an essential role in soil carbon turnover, they had been previously extremely difficult to study, which changed with the upcoming of technologies such as metagenomics (see also Falkenberg et al., 2024). These developments particularly sparked more basic research into the stabilisation and interaction dynamics of soil carbon, and models of soil carbon stabilisation were fundamentally reconsidered (Lehmann and Kleber, 2015; see, e.g. Schmidt et al., 2011).

Also contributing to the field’s growth was that more and more disciplines became interested in the topic. While soil carbon had previously been studied by traditional soil science (with a focus on physics and chemistry), other disciplines such as (microbial) ecology, biogeochemistry or climate science started to increasingly engage with the topic. The research community thus grew in size but also became more diverse and heterogeneous, with respective research being published in a broader range of journals. Since many of the disciplines newly engaging with soil carbon were seen as cutting-edge scientifically, this further pushed the prominence of the topic.

Continuously growing since the 2000s, soil carbon research experienced another upsurge with the so-called *4 per 1000* initiative that was released at COP21: ‘from the climate change perspective, *4 per 1000* … an initiative by the French government which was announced in Paris in 2015, […] really pushed soils up the agenda’ (R69). It initially claimed that a global increase of 0.4% in soil carbon stocks could compensate anthropogenic greenhouse gas emissions (Minasny et al., 2017). Next to pushing soils and soil carbon further up the public and political agenda, the initiative also gave much impetus to research on the topic (Kon Kam King et al., 2018), and the following years saw various symposia, meetings or special issues centred around the initiative’s promises. As one researcher put it, research on soil carbon ‘went nuts, it went just big when *4 per 1000* came out at COP21’ (R72).

### Bandwagon-like growth, redundancy, and an internalist focus on biophysical details

The final quotation of the previous section already indicated the strong growth, often described as bandwagon dynamic, of soil carbon research since the 2000s: ‘lots of research proposals then said, “oh, *4 per 1000*, we must understand how carbon is stabilised” and you’re just going, “Come on guys … ” It became a bandwagon, as we say’ (R72). Different researchers shared the impression that the potential of soil carbon sequestration was at times being strategically enrolled, and even exaggerated, to acquire funding, without critical engagement with the promises made. Participants also emphasised the strong growth of research output on soil carbon, noting that publications had since the early 2000s ‘just exploded’ (R62), ‘exponentially gone up in number’ (R46), and that ‘the pure volume of research on carbon, it’s just overwhelming’ (R62). Studies on soil carbon were seen as ‘very dominant … if you look at like the ten or twenty most cited or most downloaded papers in *Geoderma* [central soil science journal], for example, many, many of them will be about carbon sequestration’ (R71).

While this strong growth of soil carbon research may also be seen as positive because it was motivated by concerns about social-environmental relevance, at the time we did our interviews (2019–2022), many interviewees highlighted problematic aspects of this growth: First, participants criticised that other related and potentially equally relevant topics had, at least for some time, been pushed aside. For example, researchers described that nitrogen, and relatedly, nitrous oxide emissions, had often not been considered enough, even though being of equal (or higher) importance for sustainable soil management and greenhouse gas dynamics. One participant elaborated that ‘[carbon] is the pollutant of most interest and immediate need for addressing, but it’s not the only thing that matters. And especially in soil systems, managing for multiple outcomes requires to think about multiple factors’ (R73).

Second, researchers described how parts of the soil carbon research community had, especially after the release of the *4 per 1000* initiative, for some time become ‘very internally focused’ (R72). A lot of papers were published on how exactly carbon is stabilised in soils, how much could potentially be stored, etc. Some researchers had the impression that discussions on the topic were very much proceeding in a mode of ‘dissection’ (R35), going into ever greater details and ‘approaching this from a very technical perspective’ (R74), without considering e.g. social and economics contexts of the questions being asked.

Last, researchers noted that despite this internalist focus, soil carbon science was growing so much that it was becoming impossible ‘to keep up with all the papers being published and … to integrate all of this’ (R76). It was expressed that at times, research on the topic would get ‘a little bit redundant … ploughing the same fields over and over again’ (R74). In other words, researchers felt that the growth of soil carbon science was proceeding in a too undirected and – reflected manner, and that there was too little consideration of what kinds of knowledge in particular would still be needed in soil carbon research.

### Shifting relevancies in soil carbon research

In light of these developments, scientists described the need to re-evaluate where soil carbon science is currently standing and where it should be going. In the following, we describe some suggestions for re-orienting soil carbon research that were most prominent in our interviews and in recent publications.

First, researchers have been increasingly questioning the central focus on soil carbon at the expense of other related biophysical factors such as nitrogen (see, e.g. van Groenigen et al., 2017). These criticisms seem to have partly already led to changes in research practices. One researcher, for example, described with regards to papers solely focusing on soil carbon that ‘increasingly, if that would go to a reviewer, it would just get back saying, ‘ … even if you haven’t measured nitrous oxide you must at least include [it] in the discussion.’ So, most of the good studies now account for all the greenhouse gases.’ (R69)

Second, ever more researchers now challenge the dominant framing of soil carbon sequestration being a central solution in mitigating the climate crisis, which has informed and driven much soil carbon research in the past years (Smith, 2014; Amundson and Biardeau, 2018; Bradford et al. 2019). Like one participant put it here, they emphasise that the initial expectations in this regard were much too ambitious:

some people are saying soil carbon is going to be the saviour of the world. We don’t need to worry about climate change because all we do is sort out the soil problem and then it’s solved. That is quite obviously not true. It can be one component in solving the climate change problem, but it’s not going to solve the whole thing. We’ve got to stop emitting fossil fuels, we’ve got to decarbonise all sectors of the economy … So, it’s been overhyped a bit (R69)

Some researchers even see the framing of soil carbon sequestration as a major component in mitigating the climate crisis as even dangerous to some extent, as this runs the risk of taking ‘pressure off other mitigation practices, which may be much more important’ (R70).

At the same time, most researchers now agree that however big or small its potential contribution to climate change mitigation, increasing soil carbon is generally very beneficial for soils. One participant, for example, noted that ‘any soil carbon is great. You need to keep it in the soil, you need to get as much as you can in the soil … I’m all for soil carbon but please don’t oversell the fact that it’s somehow going to mitigate all of climate change because it won’t’ (R72). Along similar lines, many researchers noted that it is important to overcome debates about the exact potential of soil carbon storage to mitigate the climate crisis, and to instead focus on the broadly acknowledged benefits of increasing soil carbon in general (Amundson and Biardeau, 2018; Bradford et al., 2019).

Third, researchers have seen a need to shift from questions about ‘how much [carbon] can we sequester’ to ‘next questions of how should we do it, where should we do it, and things like that’ (R71). I.e. it is considered important to bring rather technical debates to a more practical and context-sensitive level – a shift that is beginning to happen since some years but still in its infancy (Beillouin et al., 2022). Rather than researching detailed numbers about how much carbon can be stored in soils, various researchers now call for more integrated modes of research producing knowledge about how to locally implement measures to increase soil carbon (Amundson and Biardeau, 2018; Poulton et al., 2018; Schlesinger and Amundson, 2019). In this regard, especially integration between natural and social sciences is considered essential: ‘you need to look a bit further away than just how is [soil carbon] now exactly mechanistically being stabilised … it’s more the step towards the non-natural sciences that needs to be made’ (R46).

Various participants pointed out that soil carbon science is in many regards already at a point where useful interventions working towards better soil management could and should be taken (Bradford et al., 2019; Vermeulen et al., 2019). As one participant elaborated:

We already know enough to take action now. We know the things that are good for increasing soil carbon. We know the things that are bad for soil carbon … we know pretty much what to do and how to do it. So, it really isn’t a matter of knowledge gaps … We maybe don’t know in enough detail the magnitude of the positive change that we’ll get in different soil types under different circumstances. But we know that it will be positive. (R69)

Researchers are thus not only calling for a different orientation of soil carbon research and changing practices of knowledge production but also for a more thorough reflection on where enough knowledge is available and different steps are thus called for.

### Fostering orientation work and a care for relevance

The calls for reconsidering the relevance of soil carbon research show the importance of constantly and actively engaging with – caring for – questions of orientation and relevance in research, rather than following pre-set goals in a bandwagon-like manner. The soil carbon case illustrates that collective engagement on the level of research communities can indeed play an important role in re-orienting research in more social-environmentally relevant ways. Yet, while many soil carbon researchers already actively try to orient research in new directions, various participants emphasised the wish that more scientists would engage in such practices and ‘stop and pause and think … as opposed to just finding out more’ (R35). One participant noted that, until now, ‘we haven’t had a real debate … there have been different opinions, but no real debates on where are we going with that research … Is this where we want to go? Do we have the tools to get the answers we need?’ (R77). In the following, we describe some practices that researchers considered to be important for engaging in orientation work and hence better caring for the relevance of their research.

#### Collective debate and development of new narratives for guiding further research

First, in light of the dynamics they had witnessed in soil carbon research in the past years, researchers described it to be important to generally make more room in research for ‘zooming out’ (R43) and tying detailed facts back to larger questions. In other words, ‘fact making must be complemented by meaning making … you start by collecting facts and understanding facts. But eventually, you have to get to the point where you say, well, what does it mean?’ (R74). Different participants voiced the impression that, all too often, scientists would ‘forget to stop, just looking down, and asking the why questions’ (R43). Researchers also emphasised the necessarily collective character of the task of integrating detailed findings back into a bigger picture, requiring coordinated exchange with others: ‘Having to place [your findings] in the larger context is definitely something that you have to be challenged with by interacting with other people’ (R58).

Participants suggested collective debates about the relevance of particular findings but also about the current orientation of soil carbon research as a whole to be particularly important to avoid an unreflected bandwagon-like growth like the one soil carbon research has seen so far. Such debates may take place at conferences, workshops, or other sorts of meetings, and potentially be facilitated by scientific organisations. Different researchers we talked to had, for example, been involved in meetings that aimed to discuss how soil carbon research should proceed after it was now increasingly agreed that the initial promises made in relation to soil carbon sequestration had been too ambitious. One researcher recalled a ‘meeting […] about two years ago where … the people really much involved in carbon sequestration … invited some more critical persons, including myself, and then we wrote this consensus paper … on basically how to implement it and how to go forward’ (R71).

Next to such meetings, researchers also described journals to be an essential platform for debates on relevance and orientation; with editorials, opinion pieces or comments as useful avenues ‘to raise issues and ask questions and … to spur things in the right direction’ (R77). They also saw editorials and opinion pieces as particularly useful for developing overarching narratives or aspirational futures for tying things together and inspiring further research – an aspect that was considered equally crucial for collectively caring for the relevance and orientation of research. One participant described the wish to ‘develop a unifying narrative or framework for latching our findings onto … an outline that takes us into future research … that seems like something that we can collectively, as a research community, aim for’ (R74). In this regard, some participants also called for more creative forms of developing narratives on relevance and orientation, for example, by drawing ‘insights in from not just physical sciences, but also the humanities and social sciences’ (R74), or even by engaging with other genres such as science fiction or poetry writing.

Whether it is in voicing particular opinions about the orientation of research on a topic or in the development of a new narrative, researchers emphasised the necessity of being able to err, in the sense of being able to leave their comfort zone by inquiring into complex phenomena and problems that do now allow for the clear statements they may be used to in their usual work, and that open up debates on the value-based character of research. Researchers nevertheless often found it essential to launch such initiates in the first place and to give new, even speculative, impulses, rather than to provide a fixed and clear-cut framework for the further development of research. One participant reflected on their own experiences with such interventions:

It's an uncomfortable position to be in when you write opinions. I wrote a couple of papers … pointing out some problems that I saw in the discipline. I got a lot of backlash from writing these things. But that's okay. I think those can be useful if they promote reflection and discussion. Even if I'm wrong, it's still good to have more discussion about where the discipline is going. (R77)

Being able to err requires certain cultural and institutional conditions of possibility, and current academic reward systems and time structures often work against this. For now, we turn to another aspect that researchers considered as essential to take better care of the relevance and direction of their work, namely the synthesis of existing knowledge.

#### Synthesising knowledge to inform discussions about the direction and relevance of research

Paying more attention to a systematic integration of gained insights was often described as essential to re-evaluate where research on a topic is currently standing and how to further proceed. Participants noted that ‘there are many hundreds of studies now on soil carbon under different management practices that can be pulled together and can enable you to make conclusions that are more general to be able to see where the difference is occurring’ (R69). Such syntheses can, first, centrally inform the crafting of narratives and collective discussions about relevance and the further development of a particular research path. Second, they may also help to evaluate whether particular new research would actually be able to deliver helpful insights. One participant stated, rather provocatively, that.

every time we raise a problem, the Pavlov reaction of researchers is, “Give us money to do research!” What we say … is: “Start with what you know! And then see if there are gaps.” If there are gaps, that’s a good basis for basic research and that’s a different approach than jumping right away to yet another program on … god knows what. (R40)

Interestingly, researchers noted that, to some extent, synthetic activities are already increasing – yet, not necessarily those kinds of synthesis that they consider most relevant. Many described a rise in meta-analyses, amongst others because these are usually highly cited and thus desired by both authors and journals, and because they are sometimes seen as a desk-based ‘easy and cheap option’ (R69). However, they remarked that meta-analyses (a quantitative and rather specialised form of synthesis) often merely ‘summarise the knowledge and they don’t contribute much’ (R71).

What was considered more useful were reviews that provide a qualitative and interpretative synthesis of existing knowledge, at best bringing together different kinds of data rather than only summarising insights in one specialised area. Researchers especially emphasised the importance of ‘forward-looking’ (R71) reviews that give impulses and suggest directions for further research.

Next to writing such qualitative and forward-looking reviews, other researchers suggested to, for example, organise dedicated synthesis workshops that ‘bring together a large group of people over a one-week period with a goal of synthesising the new data that's come out in the last 10 years or so … that's an important activity that would have to be done for soil carbon’ (R76). Some researchers were already actively engaging in such activities, for example by participating in interdisciplinary workshops that bring together different perspectives to better understand the complexities of soil organic matter stabilisation.

### Impediments to orientation work and ways forward

While many scientists saw a need for such kinds of orientation work, actually engaging in this work seemed not always easy. Synthesising or engaging in debates were described as ‘in the whole academic research wheel … not something that’s prioritised’ (R34), and as work that is not valued enough.

One hurdle was seen with regards to publication: While researchers found it essential to publish more out-of-the-box opinion papers or comments that encourage debate and suggest new directions, they felt that.

it's not always easy to publish papers like that. It's very easy to publish a paper where you measure carbon in three different treatments and say, ‘Oh, this one has more carbon than that one … ’ That kind of paper is pretty tidy, you can do nice statistical analyses and the reviewers are happy and editors are happy and you're happy because you can add another paper to your CV. (R74)

In contrast to publishing such ‘tidy’ papers, participants pointed out that it was, especially as a junior researcher, harder to make space for writing qualitative, potentially speculative reviews or opinion papers – because this requires courage and putting oneself out, but also because such output often does not count in metrics-based research evaluation. This lack of appreciation then makes it difficult to devote the time to orientation work that it would require, if taken seriously – to ‘spend four, five, six moths thinking about an issue or a problem and coming up with ideas’ (R77).

Dominant modes of research funding often neither allow to devote time to such work and rather support work that more immediately promises new discoveries and to push back scientific frontiers. Practices of synthesising or engaging in thinking and narrative-development activities are rarely supported:

It’s much easier to convincingly sell a proposal that says – “If only we knew this that’s going to help”. We’ve been saying that for a long time and people go “Yes, yes, if only we knew this, that, and the other … ” That becomes the first general agreement … we need to push back the frontiers and find out more. (R35)

Researchers moreover noted that project-based funding generally fosters the creation of ‘little bite-sized piecemeal pices of research’ (R35) rather than ‘bring[ing] groups together to synthesise … 90% of the funding goes to like an individual group doing a small project’ (R76). Through engendering such fragmentation currently dominant funding modes seem in some sense very fundamentally opposed to practicing orientation work. Given these impediments, various researchers noted an urgent need for more institutionally provided spaces that enable synthesis work and coordinated reflection on orientation and relevance.

However, researchers also saw more tacit impediments to orientation work and a greater care for relevance in the mindset and education of most academics, who are usually not used or well-trained to engage in bigger picture reflections, and ‘not very good at grappling with – “What does it mean? What now? … and where does this lead us?”’ (R74). Neither are most researchers used to thinking completely out-of-the box for directing future research in creative ways, nor to interpreting and breaking down their results in ways that might be needed for moving towards translation and application. As such, participants highlighted, creating enabling conditions for orientation work also requires changes in the education of researchers and, even more, a fundamental ‘cultural change’ (R74) in academia.

Yet, also under given conditions, researchers saw different options for engaging in more orientation work on the level of research communities. Some very senior, partly already retired researchers noted that they would consider it to be a duty of scholars like themselves, who are no longer substantially dependent on academic reward structures, to engage in synthesis and overarching reflections on the current status and future orientation of the research on a topic. As one participant described, ‘I find that a fun and intellectually rewarding thing, to … create better perspectives on the big picture issue. Younger people are under pressure to show that they can come up with their own research funds to create their own projects. So, that's a career stage issue, partly’ (R76).

Participants also suggested that journal editorial policies could have an important role in providing a platform for orientation work to happen, in that they could explicitly encourage more qualitative forward-looking reviews, opinion pieces to foster debate about the direction and relevance of research, or speculative commentaries that open up the imagination. Some researchers even toyed with more unorthodox approaches such as publishing poems or short stories. As one participant wondered, ‘what would happen if every soil science journal once a year, for example, published a short story on fiction, but fiction with the emphasis on preserving soil carbon?’ (R74).

Furthermore, it was highlighted that journal editorial policies could also play an important role not only in providing space for, but *doing* orientation work themselves, e.g. encouraging certain kinds of research and thus giving direction to the work on a topic. With regards to soil carbon, this could be to, for example, stop publishing research that was getting into redundant discussions, or papers that are ‘purely spreadsheet calculations in the absence of societal relevance’ (R76).

## Conclusions

In this paper, we take the case of soil carbon research to inquire into how research communities can contribute to adapting and re-orienting research priorities. Research is currently increasingly called upon to contribute to solving social-environmental problems. So far, attempts to govern research in this direction have mainly focused on fostering relevant research through competitive project-based research funding. The soil carbon case illustrates that competitive funding, even when focused on an issue like soil carbon that is broadly considered relevant, can only to some extent, and only on a short-term basis, incentivise research that addresses social-environmental problems. While soil carbon research experienced a strong increase in research volume, its proponents at some point started criticising its bandwagon-like growth, impeding a discussion about other kinds of climate-relevant soil research. This emphasises the importance of not tick-boxing relevance at a single point in time but instead adopting a processual understanding of relevance (Mol, 2008; Fisher and Schuurbiers, 2013; Stilgoe et al. 2013, Martin, Myers, and Viseu, 2015; Savransky, 2016).

We argue that sustainable orientation towards relevance should not be an isolated event but is most effective when it follows key characteristics of a care relationship to social-environmental problems (Mol, 2008; Martin, Myers, and Viseu, 2015; Haraway, 2016): in this conceptual perspective, orientation work in the sense of caring for relevance needs to be *open-ended and responsive*, *collective*, requires certain *conditions of possibility* and requires to pay *attention to value-based questions*.

Our first emphasis is on the *open-ended, responsive* character of caring for relevance. We have described how soil carbon science has over time to a certain extent become captured in bandwagon-like growth dynamics. Researchers did not consider more knowledge on soil carbon as productive contribution to relevance anymore, arguing that while it is sufficiently established that soil carbon is an important factor in climate change dynamics, other important factors have been relatively under-researched. This shows how the relevance of a research topic, in the sense of its potential for informing problem definitions and related solutions, is not fixed but changes over time, in relation to the state of knowledge on a topic, and in relation to how problems are societally addressed at different points in time. Other kinds of soil research beyond soil carbon storage in a narrow sense appear be more relevant to the community today, such as research on other greenhouse gases related to soils, more interdisciplinary approaches that focus on interrelations between soil properties, or research related to factors that support societal and economic challenges related to changes in soil management (Falkenberg et al. 2024; Chabbi et al. 2021). This shows that caring for relevance is always open-ended and in transition, and implies to stay responsive to how the state of knowledge on a topic develops, but also to societal and environmental changes. While for example the concept of responsible research and innovation also considers the dimension of responsiveness, it is mainly concerned with responsiveness to societal and environmental problems, or the concerns of stakeholders, whereas our analysis illustrates that it is essential to also cultivate responsiveness to scientific developments (cf. Stilgoe et al. 2013).

The second characteristic we want to emphasise is that caring for relevance is a *collective* practice. We have shown how the related research communities have been collectively deliberating the relevance of soil carbon research in several ways. For doing so, they used formats that are common to academic work such as special issues, co-authored papers and conferences, but also formats that are less common and less valued in terms of academic performance, such as policy related initiatives, reports and events. These practices are collective as they usually involve, or start from, a group of like-minded researchers or because they entail practices that are necessarily collectivising (such as synthesis). To become effective in steering the priorities of a research field to some extent, such caring for relevance needs broader agreements within a field, and new narratives about the relevance of research that are ideally shared by a broad social basis. Researchers emphasised the importance of narratives to achieve this, and argued for the importance of narrative experimentation, also in formats such as short stories or poems. While soil researchers in our sample were typically quite motivated to engaging in these activities, they strongly emphasised that there were little reward structures for doing so in their everyday environment.

Our third characteristic thus entails *conditions of possibility*, i.e. a need for enabling conditions in research cultures related to the above characteristics: rather than focusing on productivity within a fixed time period, researchers require conditions that allow them and their work to be flexible and open-ended enough to react to unforeseen developments (Sigl and Fochler, 2025). They need conditions that encourage not individuation and competition but collectivity and coordination. Furthermore, there needs to be more emphasis on processes of deliberation of what (should) count as valuable work in academia and what it means to advance science.

In relation to our empirical case, we emphasise the importance of valuing research output that is dedicated to synthesising existing knowledge, that engages in discussions about the relevance of research orientation, or that creatively develops new narratives and imaginaries about relevance. To avoid career risks particularly for untenured researchers, it seems crucial to recognise and reward such practices in evaluation and career contexts. Similar arguments for such institutional change have been brought forward in relation to strengthening societal responsibility of individual researchers (‘supportive environments’, Kuhlmann et al. 2016; ‘responsibility conditions’, Felt, 2017). Our study makes a notable extension to these arguments, namely that similar conditions also seem to be crucial for fostering collective orientation work in broader research fields.

Fourth, *attention to value-based questions* is crucial in caring for relevance. What knowledge is relevant is ultimately also a question linked to socio-political visions of desirable futures, and to commitments to political aims in the present (Granjou and Arpin, 2015). Which knowledge is relevant for addressing the climate crisis for example depends on assumptions on who is responsible for this crisis, who should act on it in which ways, whose livelihoods are to be protected or may be endangered, and how humanity should relate to the planet sustaining it in a broader sense. These are big questions, but any statement on the relevance of knowledge ultimately entails positionings in such larger value-based debates. In this paper, we have not been able to trace this empirically, differently from the three characteristics discussed above. Elsewhere (Sigl et al. 2023) we have done related work on the broader field of the soil sciences in a longer temporal perspective. We are convinced that analysing the value-based dimension of orientation work as caring for relevance will be of key importance for further work interested in processes related to orientation work.

In a world in which relevant knowledge is seen as crucial for the future of humanity and our planet and in which what is perceived as relevant is crucial in orienting the production of knowledge, actively caring for relevance is of key importance for scientific communities. Collective orientation work, as we describe in this paper, is crucial in this. STS research can and should help scientists and their communities to more reflexively engage in this work, and it should inform how academic institutions and research communities can structurally support orientation work on a continual basis. As STS researchers, this collectively challenges us to invest in orientation work of our own (see Kaltenbrunner et al. 2022 for an example), in order to care for the relevance of STS in times of planetary crises.

## Notes

1 https://4p1000.org (accessed 17.01.2023)

2 https://research-and-innovation.ec.europa.eu/funding/funding-opportunities/funding-programmes-and-open-calls/horizon-europe/eu-missions-horizon-europe/soil-health-and-food_en (accessed 17.01.2023)

3 For more information on the framework of this project, please see: https://rri.univie.ac.at/forschung/forschungsprojekte/valuing-being-and-knowing-in-research-practices/ (25.11.2024)

4 Soil carbon consists of both inorganic and organic carbon. The latter, in turn, makes up the largest component of soil organic matter. Partly, similar kinds of research have been carried out under the labels of soil carbon and soil organic matter (Smith et al. 2018). Despite this sometimes overlapping use of terminology, we do in the following employ the term soil carbon as the one that has gained particular prominence since some decades.

5 Participants in our research projects are cited using a pseudonym in the form of ‘Rx’, x being a number chronologically assigned to researchers participating in interviews or group discussions.
